# P-2047. Reducing Broad-Spectrum Antibiotic Use through a Multi-Modal Educational Campaign in Patients with Penicillin Allergy

**DOI:** 10.1093/ofid/ofaf695.2211

**Published:** 2026-01-11

**Authors:** Srishti Chhabra, Hui Hiong Chen, Priscillia Lye, Jia En Wu, Fathima Rofina Farveen Mohamed Nasar, Geraldine Foo, Amelia Santosa, Sophia Archuleta, Nares Smitasin

**Affiliations:** National University Health System, Singapore, Singapore; NUH, Singapore, Not Applicable, Singapore; Alexandra Hospital, Singapore, Singapore, Not Applicable, Singapore; National University Hospital, Singapore, Singapore, Not Applicable, Singapore; National University Hospital, Singapore, Singapore, Not Applicable, Singapore; National University Hospital Singapore, Singapore, Not Applicable, Singapore; National University Hospital, Singapore, Singapore, Not Applicable, Singapore; NUHS, Singapore, Not Applicable, Singapore; National University Hospital, Singapore, Singapore, Not Applicable, Singapore

## Abstract

**Background:**

Penicillin allergies (PA) are over-reported, resulting in the use of less preferred broad-spectrum antibiotics with higher rates of treatment failure. Cephalosporins are infrequently used in patients with reported PA due to fears of cross-reactivity. The aim of our study was to utilize the PEN-FAST score to identify patients with low-risk of PA, to increase cephalosporin use in this patient group.

**Methods:**

This was a retrospective study done in four departments at a tertiary hospital in Singapore from July-December 2024. Patients aged > 18 years with PA who were admitted for > 48 hours, required antibiotics and had a PEN-FAST score of 0-2 were included. Exclusion criteria was allergy to cephalosporins, clinical instability and patients in whom the use of broad-spectrum (eg carbapenem) or second-line antibiotics was appropriate. We introduced a workflow to guide physicians in utilizing the PEN-FAST to assess which patients with PA were suitable for cephalosporin use (Figure 1). A multi-modal approach was used to disseminate this workflow through the hospital intranet, department meetings and resident teaching sessions. Pharmacists utilized the hospital IT system to identify patients on second-line antibiotics who could receive cephalosporins. We compared percentage of patients who appropriately received cephalosporins and days of less preferred antibiotic use pre-and post-intervention.

**Results:**

A total of 97 patients met the study criteria with 30 identified pre-intervention and 67 identified post-intervention. The use of cephalosporins amongst patients with PA increased from 46.7% (16/30) to 76.1% (51/67) post intervention (Figure 2). There was no significant difference in age, gender, PEN-FAST score, number of drug class allergies amongst those who were prescribed cephalosporins and those who were not (Table 1). Patients who were prescribed cephalosporins received a shorter duration of broad spectrum/second-line antibiotics (1 day (IQR 0-3.9) vs 3.9 days (IQR 3-5); p< 0.01). No allergic reactions were reported in those who were prescribed cephalosporins.

**Conclusion:**

The PEN-FAST score can be utilized to safely reduce broad-spectrum antibiotic use. Further work is required to assess its long-term impact on colonization and infection with multi-drug-resistant organisms.

**Disclosures:**

All Authors: No reported disclosures

